# Crystal structure of bis­[*cis*-(1,4,8,11-tetra­aza­cyclo­tetra­deca­ne-κ^4^ *N*)bis(thio­cyanato-κ*N*)chrom­ium(III)] dichromate monohydrate from synchrotron X-ray diffraction data

**DOI:** 10.1107/S2056989016020120

**Published:** 2017-01-01

**Authors:** Dohyun Moon, Masahiro Takase, Takashiro Akitsu, Jong-Ha Choi

**Affiliations:** a Pohang Accelerator Laboratory, POSTECH, Pohang 37673, Republic of Korea; b Department of Chemistry, Tokyo University of Science, 1-3 Kagurazaka, Shinjuku-ku, Tokyo 162-8601, Japan; c Department of Chemistry, Andong National University, Andong 36729, Republic of Korea

**Keywords:** crystal structure, cyclam, chrom­ium(III) complex, thio­cyanate ligand, *cis*-V conformation, dichromate anion, hydrogen bonding, synchrotron radiation

## Abstract

The structure of the complex salt, *cis*-[Cr(NCS)_2_(cyclam)]_2_[Cr_2_O_7_]·H_2_O (cyclam = 1,4,8,11-tetra­aza­cyclo­tetra­decane, C_10_H_24_N_4_), has been determined from synchrotron data. The asymmetric unit comprises of one [Cr(NCS)_2_(cyclam)]^+^ cation, one half of a Cr_2_O_7_^2−^ anion (completed by inversion symmetry) and one half of a water mol­ecule (completed by twofold rotation symmetry). The Cr^III^ ion is coordinated by the four cyclam N atoms and by two N atoms of *cis*-arranged thio­cyanate anions, displaying a distorted octa­hedral coordination sphere. The Cr—N(cyclam) bond lengths are in the range 2.080 (2) to 2.097 (2) Å while the average Cr—N(NCS) bond length is 1.985 (4) Å. The macrocyclic cyclam moiety adopts the *cis*-V conformation. The bridging O atom of the dichromate anion is disordered around an inversion centre, leading to a bending of the Cr—O—Cr bridging angle [157.7 (3)°]; the anion has a staggered conformation. The crystal structure is stabilized by inter­molecular hydrogen bonds involving the cyclam N—H groups and water O—H groups as donor groups, and the O atoms of the Cr_2_O_7_^2−^ anion and water mol­ecules as acceptor groups, giving rise to a three-dimensional network.

## Abstract 2

The asymmetric unit of the title compound comprises of one complex cation, one half of a Cr_2_O_7_^2−^ anion and one half of a water mol­ecule. The Cr^III^ ion has a distorted octa­hedral coordination by four N atoms of the cyclam ligand and by two N-bonded NCS groups in *cis* positions; the conformation of the dichromate anion is staggered.

## Chemical context   

Recently, it has been established that cyclam (1,4,8,11-tetra­aza­cyclo­tetra­decane, C_10_H_24_N_4_) derivatives and their complexes can exhibit anti-HIV effects or stimulate the activity of stem cells from bone marrow (Ronconi & Sadler, 2007 ▸; De Clercq, 2010 ▸; Ross *et al.*, 2012 ▸). Cyclam has a moderately flexible structure and can adopt both planar (*trans*) and folded (*cis*) conformations (Poon & Pun, 1980 ▸). There are five configurational *trans* isomers for the macrocycle, which differ in the chirality of the *sec*-NH sites (Choi, 2009 ▸). The *trans*-I, *trans*-II and *trans*-V configurations also can fold to form *cis*-I, *cis*-II and *cis*-V isomers, respectively (Subhan *et al.*, 2011 ▸). The configuration of the macrocyclic ligand and the influence of the counter-anion are important factors in developing new highly effective anti*-*HIV drugs.

The dichromate anion is environmentally important due to its high toxicity (Yusof & Malek, 2009 ▸), and its use in industrial processing (Goyal *et al.*, 2003 ▸). Since counter-anionic species play an important role in coordination chemistry (Martínez-Máñez & Sancenón, 2003 ▸; Fabbrizzi & Poggi, 2013 ▸), it may be possible that the [Cr(NCS)_2_(cyclam)]^+^ cation is suitable to bind specifically to an oxoanion. In this context, we report here on the synthesis of a new chromium(III)–dichromate salt, [Cr(NCS)_2_(cyclam)]_2_(Cr_2_O_7_)·H_2_O, (I), and its structural characterization by synchrotron single-crystal X-ray diffraction.
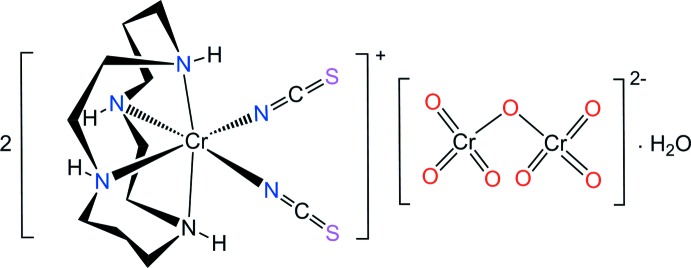


## Structural commentary   

**Figure 1:**
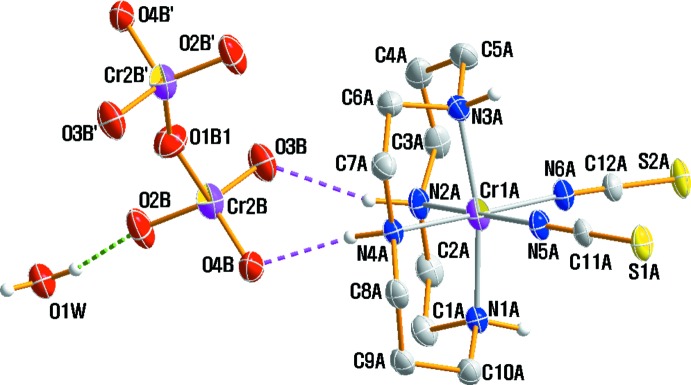
The mol­ecular components in the structure of (I) with displacement ellipsoids drawn at the 30% probability level. Only one orientation of the disordered anion is shown; primed atoms are related by symmetry code (−*x*, −*y* + 1, −*z* − 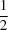). Dashed lines represent hydrogen bonds.

Fig. 1 ▸ displays the mol­ecular components of (I). The structure is another example of a [Cr(NCS)_2_(cyclam)]^+^ cation (Friesen *et al.*, 1997 ▸; Moon *et al.*, 2013 ▸) but with a different counter-anion. The asymmetric unit comprises of one [Cr(NCS)_2_(cyclam)]^+^ cation, one half of a Cr_2_O_7_^2−^ anion (completed by inversion symmetry) and one half of a water mol­ecule (completed by twofold rotation symmetry). In the complex cation, the Cr^III^ ion is coordinated by the N atoms of the cyclam ligand in the folded conformation. The nitro­gen atoms of two NCS^−^ ligands coordinate to the chromium atoms in a *cis* arrangement. The cyclam moiety adopts the *cis*-V (*anti*–*anti*) conformation (Subhan *et al.*, 2011 ▸). The Cr—N(cyclam) bond lengths are in the range 2.080 (2) to 2.097 (2) Å, in good agreement with those determined in related structures, namely *cis*-[Cr(NCS)_2_(cyclam)]SCN [2.0851 (14)–2.0897 (14) Å; Moon *et al.*, 2013 ▸], *cis-*[Cr(N_3_)_2_(cyclam)]ClO_4_ [2.069 (3)–2.103 (3) Å; Meyer *et al.*, 1998 ▸], *cis*-[Cr(ONO)_2_(cyclam)]NO_2_ [2.0874 (16)–2.0916 (15) Å; Choi *et al.*, 2004*a* ▸], [Cr(acac)(cyclam)](ClO_4_)_2_·0.5H_2_O [2.070(5–2.089 (5) Å, acac = acetyl­acetonate; Subhan *et al.*, 2011 ▸] or *cis*-[CrCl_2_(cyclam)][Cr(ox)(cyclam)](ClO_4_)_2_ [2.075 (5)–2.096 (5) Å; Moon & Choi, 2016*a* ▸]. The Cr—N(cyclam) bond lengths with co-ligands in *cis* orientations are slightly longer than those found in *trans-*[Cr(NCS)_2_(cyclam)]ClO_4_ [2.046 (2)–2.060 (2) Å; Friesen *et al.*, 1997 ▸], *trans*-[Cr(ONO)_2_(cyclam)]BF_4_ [2.064 (4)–2.073 (4) Å; De Leo *et al.*, 2000 ▸], *trans-*[Cr(NH_3_)_2_(cyclam)][ZnCl_4_]Cl·H_2_O [2.0501 (15)–2.0615 (15) Å; Moon & Choi, 2016*b* ▸] or *trans*-[Cr(nic-O)_2_(cyclam)]ClO_4_ [2.058 (4) – 2.064 (4) Å, nic-O = O-coordinating nicotinate; Choi, 2009 ▸]. The two Cr—N(NCS) bond lengths in (I) average to 1.985 (4) Å and are close to the values found in *cis*-[Cr(NCS)_2_(cyclam)]NCS [1.996 (15) Å; Moon *et al.*, 2013 ▸], *cis*-[Cr(NCS)_2_(cyclam)]ClO_4_ [1.981 (4)–1.998 (4) Å; Friesen *et al.*, 1997 ▸], *trans-*[Cr(NCS)_2_(cyclam)]_2_[ZnCl_4_] [1.995 (6) Å; Moon *et al.*, 2015*a* ▸] or *trans*-[Cr(NCS)_2_(Me_2_tn)_2_]SCN·0.5H_2_O [1.983 (2)–1.990 (2) Å; Choi & Lee, 2009 ▸]. The five- and six-membered chelate rings of the cyclam ligand adopt *gauche* and stable chair conformations, respectively. The folded angle [96.05 (8)°] of cyclam is comparable to the values of 98.55 (2), 97.17 (5), 97.03 (2), 95.09 (9), 94.51 (2) and 92.8 (2)° in [Cr(ox)(cyclam)]ClO_4_, *cis*-[Cr(NCS)_2_(cyclam)]SCN, [Cr(acac)(cyclam)](ClO_4_)_2_·0.5H_2_O, *cis*-[Cr(ONO)_2_(cyclam)]NO_2_, *cis-*[Cr(N_3_)_2_(cyclam)]ClO_4_ and *cis*-[Cr(cyclam)Cl_2_]Cl, respectively (Choi *et al.*, 2004*b* ▸; Moon *et al.*, 2013 ▸; Subhan *et al.*, 2011 ▸; Choi *et al.*, 2004*a* ▸; Meyer *et al.*, 1998 ▸; Forsellini *et al.*, 1986 ▸, respectively).

The two N-bound thio­cyanate anions are almost linear, with N—C—S angles of 178.8 (2) and 179.0 (3)°. The bridging O atom of the Cr_2_O_7_^2−^ anion is positionally disordered over an inversion centre, giving rise to a bending of the Cr2*B*—O1*B*—Cr2*B*(−*x* + 1, −*y* + 1, −*z* + 1) angle [157.7 (3)°]. The Cr_2_O_7_^2−^ anion in (I) has a staggered conformation while a nearly eclipsed conformation is observed in ionic compounds K_2_Cr_2_O_7_, Rb_2_Cr_2_O_7_ and (C_3_H_5_N_2_)(NH_4_)[Cr_2_O_7_] (Brandon & Brown, 1968 ▸; Löfgren, 1971 ▸; Zhu, 2012 ▸). The conformation of the dichromate anion is influenced by the charge and size of the counter-cation (Moon *et al.*, 2015*b* ▸; Moon & Choi, 2016 ▸). The O—Cr2*B*—O bond angles range from 102.3 (3) to 119.5 (2)°; the terminal Cr2*B*—O bond lengths vary from 1.596 (2) to 1.612 (2) Å, with a mean terminal Cr2*B*—O bond length of 1.604 (12) Å. The bridging Cr2*B*—O1*B* bond has a length of 1.746 (9) Å. These values are comparable to those reported for the anions in the structures of [Cr(urea)_6_](Cr_2_O_7_)Br·H_2_O (Moon *et al.*, 2015*b* ▸) or [CrCl_2_(tn)_2_]_2_(Cr_2_O_7_) (tn = propane-1,3-di­amine; Moon & Choi, 2016 ▸). A further distortion of the anion is due to its involvement in hydrogen-bonding inter­actions with water mol­ecule and complex cation (see *Supra­molecular features*).

## Supra­molecular features   

Two O—H⋯O hydrogen bonds link the water mol­ecule to neighboring Cr_2_O_7_^2−^ anions while N—H⋯O hydrogen bonds inter­connect [Cr(NCS)_2_(cyclam)]^+^ cations with both the anions and water mol­ecules (Table 1 ▸; Figs. 1 ▸ and 2 ▸) . An extensive array of these contacts generates a three-dimensional network of mol­ecules stacked along the *c*-axis.

## Database survey   

A search of the Cambridge Structural Database (Version 5.37, Feb 2016 with two updates; Groom *et al.*, 2016 ▸) gave 17 hits for a *cis*-[Cr*L*_2_(C_10_H_24_N_4_)]^+^ unit.

## Synthesis and crystallization   

Cyclam was purchased from Stream Chemicals and used as provided. All chemicals were reagent-grade materials and used without further purification. The starting material, *cis*-[Cr(NCS)_2_(cyclam)]SCN was prepared according to a literature protocol (Ferguson & Tobe, 1970 ▸). The thio­cyanate salt (0.513 g) was dissolved in 15 mL water at 347 K. The filtrate was added to 5 mL of water containing solid K_2_Cr_2_O_7_ (0.02 g). The resulting solution was evaporated slowly at room temperature until formation of crystals. The obtained block-like orange crystals of the dichromate salt were washed with small amounts of 2-propanol and dried in air before collecting the synchrotron data. Elemental analysis calculated for [Cr(NCS)_2_(C_10_H_24_N_4_)]_2_(Cr_2_O_7_)·H_2_O: C, 29.69; H, 5.19; N, 17.31%; found C, 29.84; H, 4.90; N, 17.28%.

## Refinement   

Crystal data, data collection and structure refinement details are summarized in Table 2 ▸. All H atoms were placed in geometrically idealized positions and constrained to ride on their parent atoms, with C—H = 0.98 Å and N—H = 0.99 Å, and with *U*_iso_(H) values of 1.2*U*_eq_ of the parent atoms. The hydrogen atom of the solvent water mol­ecule was assigned based on a difference Fourier map, and the O—H distance and the H—O—H angle were restrained [0.84 (1) Å, 136 (2)°]. The bridging oxygen atom of the dichromate anion is positionally disordered around an inversion centre and consequently was refined with half-occupancy.

## Supplementary Material

Crystal structure: contains datablock(s) I. DOI: 10.1107/S2056989016020120/wm5351sup1.cif

Structure factors: contains datablock(s) I. DOI: 10.1107/S2056989016020120/wm5351Isup2.hkl

CCDC reference: 1523266

Additional supporting information: crystallographic information; 3D view; checkCIF report

## Figures and Tables

**Figure 2:**
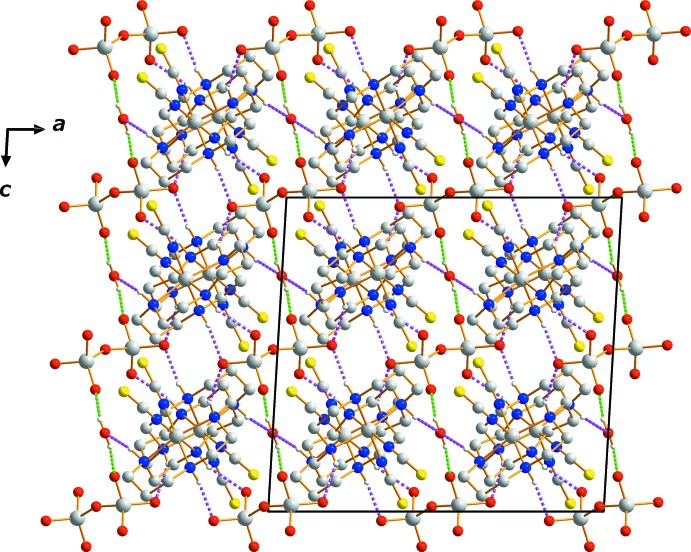
The crystal packing in compound (I), viewed perpendicular to the *ac* plane. Dashed lines represent O—H⋯O (green) and N—-H⋯O (pink) hydrogen-bonding inter­actions.

**Table 1:** Hydrogen-bond geometry (Å, °)

*D*—H⋯*A*	*D*—H	H⋯*A*	*D*⋯*A*	*D*—H⋯*A*
N1*A*—H1*A*⋯O1*W* ^i^	0.99	2.15	3.089 (3)	157
N2*A*—H2*A*⋯O3*B*	0.99	2.17	3.127 (3)	163
N3*A*—H3*A*⋯O4*B* ^ii^	0.99	2.10	2.953 (3)	143
N4*A*—H4*A*⋯O4*B*	0.99	1.99	2.904 (3)	152
O1*W*—H1*OW*⋯O2*B*	0.84 (1)	2.24 (1)	3.052 (3)	164 (2)

Symmetry codes: (i) 
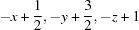
; (ii) 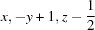.

**Table 2:** Experimental details

Crystal data	
Chemical formula	[Cr(NCS)_2_(C_10_H_24_N_4_)]_2_[Cr_2_O_7_]·H_2_O
*M*_r_	971.00
Crystal system, space group	Monoclinic, *C*2/*c*
Temperature (K)	243
*a*, *b*, *c* (Å)	16.044 (2), 16.221 (2), 15.041 (2)
β (°)	93.335 (3)
*V* (Å^3^)	3907.8 (9)
*Z*	4
Radiation type	Synchrotron, λ = 0.620 Å
μ (mm^−1^)	0.92
Crystal size (mm)	0.04 × 0.03 × 0.02

Data collection	
Diffractometer	ADSC Q210 CCD area detector
Absorption correction	Empirical (using intensity measurements) (*HKL3000sm *SCALEPACK**; Otwinowski & Minor, 1997 ▸)
*T*_min_, *T*_max_	0.799, 1.000
No. of measured, independent and observed [*I* > 2σ(*I*)] reflections	11326, 5767, 4156
*R*_int_	0.018
(sin θ/λ)_max_ (Å^−1^)	0.707

Refinement	
*R*[*F* ^2^ > 2σ(*F* ^2^)], *wR*(*F* ^2^), *S*	0.046, 0.148, 1.06
No. of reflections	5767
No. of parameters	244
No. of restraints	3
H-atom treatment	H atoms treated by a mixture of independent and constrained refinement
Δρ_max_, Δρ_min_ (e Å^−3^)	1.07, −0.73

Computer programs: *PAL BL2D-SMDC* (Shin *et al.*, 2016 ▸), *HKL3000sm* (Otwinowski & Minor, 1997 ▸), *SHELXT2014* (Sheldrick, 2015*a* ▸), *SHELXL2016* (Sheldrick, 2015*b* ▸), *DIAMOND 4* (Putz & Brandenburg, 2014 ▸), *publCIF* (Westrip,2010 ▸).

## supplementary crystallographic information

### Crystal data

**Table:**

[Cr(NCS)_2_(C_10_H_24_N_4_)]_2_[Cr_2_O_7_]·H_2_O	*F*(000) = 2008
*M**_r_* = 971.00	*D*_x_ = 1.650 Mg m^−^^3^
Monoclinic, *C*2/*c*	Synchrotron radiation, λ = 0.620 Å
*a* = 16.044 (2) Å	Cell parameters from 51334 reflections
*b* = 16.221 (2) Å	θ = 0.4–33.6°
*c* = 15.041 (2) Å	µ = 0.92 mm^−^^1^
β = 93.335 (3)°	*T* = 243 K
*V* = 3907.8 (9) Å^3^	Block, orange
*Z* = 4	0.04 × 0.03 × 0.02 mm

### Data collection

**Table:**

ADSC Q210 CCD area detector diffractometer	4156 reflections with *I* > 2σ(*I*)
Radiation source: PLSII 2D bending magnet	*R*_int_ = 0.018
ω scan	θ_max_ = 26.0°, θ_min_ = 1.6°
Absorption correction: empirical (using intensity measurements) (*HKL3000sm Scalepack*; Otwinowski & Minor, 1997)	*h* = −22→22
*T*_min_ = 0.799, *T*_max_ = 1.000	*k* = −22→22
11326 measured reflections	*l* = −21→21
5767 independent reflections	

### Refinement

**Table:**

Refinement on *F*^2^	Hydrogen site location: mixed
Least-squares matrix: full	H atoms treated by a mixture of independent and constrained refinement
*R*[*F*^2^ > 2σ(*F*^2^)] = 0.046	*w* = 1/[σ^2^(*F*_o_^2^) + (0.0961*P*)^2^] where *P* = (*F*_o_^2^ + 2*F*_c_^2^)/3
*wR*(*F*^2^) = 0.148	(Δ/σ)_max_ = 0.001
*S* = 1.06	Δρ_max_ = 1.07 e Å^−^^3^
5767 reflections	Δρ_min_ = −0.73 e Å^−^^3^
244 parameters	Extinction correction: SHELXL-2016/6 (Sheldrick 2015), Fc^*^=kFc[1+0.001xFc^2^λ^3^/sin(2θ)]^-1/4^
3 restraints	Extinction coefficient: 0.0074 (7)

### Special details

**Table:**

Geometry. All esds (except the esd in the dihedral angle between two l.s. planes) are estimated using the full covariance matrix. The cell esds are taken into account individually in the estimation of esds in distances, angles and torsion angles; correlations between esds in cell parameters are only used when they are defined by crystal symmetry. An approximate (isotropic) treatment of cell esds is used for estimating esds involving l.s. planes.

### Fractional atomic coordinates and isotropic or equivalent isotropic displacement parameters (Å^2^)

**Table:**

	*x*	*y*	*z*	*U*_iso_*/*U*_eq_	Occ. (<1)
Cr1A	0.21438 (2)	0.57578 (2)	0.25925 (2)	0.03091 (13)	
S1A	0.04950 (5)	0.36485 (4)	0.11771 (4)	0.04880 (19)	
S2A	0.10063 (7)	0.74359 (5)	0.02581 (6)	0.0745 (3)	
N1A	0.12183 (13)	0.61482 (13)	0.34086 (12)	0.0382 (4)	
H1A	0.079016	0.642089	0.300936	0.046*	
N2A	0.27533 (14)	0.68147 (13)	0.31024 (13)	0.0434 (5)	
H2A	0.316020	0.664275	0.358709	0.052*	
N3A	0.32354 (14)	0.54328 (15)	0.19902 (14)	0.0446 (5)	
H3A	0.306385	0.502794	0.152215	0.054*	
N4A	0.25948 (13)	0.49636 (12)	0.35970 (12)	0.0346 (4)	
H4A	0.288918	0.530211	0.406434	0.041*	
N5A	0.15160 (14)	0.48029 (13)	0.20628 (13)	0.0413 (5)	
N6A	0.17022 (15)	0.64396 (14)	0.15798 (14)	0.0438 (5)	
C1A	0.15574 (19)	0.68028 (17)	0.40197 (18)	0.0494 (6)	
H1A1	0.188173	0.655601	0.452403	0.059*	
H1A2	0.110059	0.712466	0.425097	0.059*	
C2A	0.2102 (2)	0.73442 (17)	0.34991 (19)	0.0529 (7)	
H2A1	0.237031	0.776231	0.388958	0.063*	
H2A2	0.176737	0.762617	0.302565	0.063*	
C3A	0.3207 (2)	0.73028 (18)	0.24429 (19)	0.0538 (7)	
H3A1	0.341165	0.781628	0.272119	0.065*	
H3A2	0.282160	0.744693	0.193779	0.065*	
C4A	0.39357 (19)	0.6825 (2)	0.2110 (2)	0.0590 (8)	
H4A1	0.428513	0.663492	0.262585	0.071*	
H4A2	0.427404	0.720062	0.176952	0.071*	
C5A	0.3701 (2)	0.6088 (2)	0.15325 (18)	0.0562 (7)	
H5A1	0.335888	0.627816	0.101176	0.067*	
H5A2	0.421255	0.584763	0.131733	0.067*	
C6A	0.37777 (18)	0.49700 (19)	0.26556 (18)	0.0504 (6)	
H6A1	0.409094	0.535645	0.304869	0.061*	
H6A2	0.417792	0.463030	0.235079	0.061*	
C7A	0.32269 (18)	0.44260 (17)	0.31975 (18)	0.0461 (6)	
H7A1	0.294855	0.400899	0.281353	0.055*	
H7A2	0.356505	0.414318	0.366772	0.055*	
C8A	0.19634 (16)	0.44486 (15)	0.40364 (16)	0.0410 (5)	
H8A1	0.225168	0.408506	0.447454	0.049*	
H8A2	0.166940	0.410082	0.358709	0.049*	
C9A	0.13381 (18)	0.49627 (17)	0.44937 (16)	0.0446 (6)	
H9A1	0.164157	0.532275	0.492398	0.053*	
H9A2	0.098970	0.459365	0.483051	0.053*	
C10A	0.07670 (17)	0.54960 (17)	0.38902 (17)	0.0441 (6)	
H10A	0.046651	0.514150	0.345211	0.053*	
H10B	0.035225	0.575948	0.425015	0.053*	
C11A	0.10820 (16)	0.43251 (14)	0.16879 (14)	0.0354 (5)	
C12A	0.14135 (15)	0.68562 (15)	0.10176 (15)	0.0376 (5)	
Cr2B	0.43256 (3)	0.58043 (3)	0.52084 (3)	0.04068 (14)	
O1B1	0.5133 (5)	0.5087 (6)	0.5160 (6)	0.0703 (17)	0.25
O1B2	0.5133 (5)	0.5087 (6)	0.5160 (6)	0.0703 (17)	0.25
O2B	0.46377 (19)	0.62450 (18)	0.61164 (14)	0.0817 (8)	
O3B	0.43108 (16)	0.64288 (14)	0.43819 (14)	0.0686 (6)	
O4B	0.33924 (14)	0.54580 (18)	0.52960 (14)	0.0737 (7)	
O1W	0.500000	0.75939 (19)	0.750000	0.0587 (8)	
H1OW	0.483 (2)	0.7293 (10)	0.7074 (11)	0.088*	

### Atomic displacement parameters (Å^2^)

**Table:**

	*U*^11^	*U*^22^	*U*^33^	*U*^12^	*U*^13^	*U*^23^
Cr1A	0.0380 (2)	0.0291 (2)	0.02458 (18)	−0.00533 (14)	−0.00667 (13)	0.00188 (12)
S1A	0.0635 (4)	0.0365 (3)	0.0439 (3)	−0.0122 (3)	−0.0183 (3)	−0.0014 (3)
S2A	0.1159 (8)	0.0473 (4)	0.0554 (5)	0.0173 (5)	−0.0380 (5)	0.0057 (4)
N1A	0.0448 (11)	0.0365 (11)	0.0325 (9)	−0.0010 (9)	−0.0040 (8)	0.0010 (8)
N2A	0.0535 (12)	0.0377 (11)	0.0372 (10)	−0.0140 (9)	−0.0116 (9)	0.0014 (9)
N3A	0.0447 (12)	0.0561 (13)	0.0328 (10)	−0.0070 (10)	0.0005 (8)	0.0015 (9)
N4A	0.0430 (10)	0.0318 (9)	0.0282 (8)	−0.0009 (8)	−0.0035 (7)	0.0029 (7)
N5A	0.0519 (12)	0.0368 (11)	0.0337 (9)	−0.0103 (9)	−0.0092 (8)	−0.0011 (8)
N6A	0.0552 (13)	0.0402 (12)	0.0343 (10)	−0.0057 (9)	−0.0104 (9)	0.0065 (8)
C1A	0.0675 (18)	0.0390 (13)	0.0409 (13)	−0.0006 (12)	−0.0021 (12)	−0.0068 (11)
C2A	0.075 (2)	0.0344 (13)	0.0481 (14)	−0.0057 (13)	−0.0049 (13)	−0.0066 (11)
C3A	0.0632 (18)	0.0456 (15)	0.0511 (15)	−0.0241 (13)	−0.0085 (13)	0.0059 (12)
C4A	0.0543 (17)	0.071 (2)	0.0515 (15)	−0.0223 (15)	−0.0041 (13)	0.0133 (14)
C5A	0.0542 (16)	0.072 (2)	0.0432 (14)	−0.0119 (15)	0.0063 (12)	0.0119 (14)
C6A	0.0456 (14)	0.0634 (17)	0.0422 (13)	0.0067 (13)	0.0018 (11)	0.0010 (13)
C7A	0.0494 (14)	0.0466 (14)	0.0418 (13)	0.0099 (12)	−0.0011 (11)	0.0019 (11)
C8A	0.0526 (14)	0.0347 (12)	0.0351 (11)	−0.0056 (10)	−0.0029 (10)	0.0099 (9)
C9A	0.0524 (15)	0.0485 (14)	0.0329 (11)	−0.0050 (12)	0.0027 (10)	0.0081 (10)
C10A	0.0446 (13)	0.0479 (14)	0.0397 (12)	−0.0025 (11)	0.0004 (10)	0.0054 (11)
C11A	0.0472 (13)	0.0308 (11)	0.0273 (10)	−0.0001 (9)	−0.0068 (9)	0.0033 (8)
C12A	0.0463 (13)	0.0354 (12)	0.0300 (10)	−0.0024 (10)	−0.0068 (9)	−0.0036 (9)
Cr2B	0.0457 (2)	0.0434 (3)	0.0319 (2)	0.00024 (17)	−0.00628 (16)	0.00294 (15)
O1B1	0.060 (5)	0.063 (4)	0.087 (6)	0.014 (3)	0.000 (3)	−0.009 (4)
O1B2	0.060 (5)	0.063 (4)	0.087 (6)	0.014 (3)	0.000 (3)	−0.009 (4)
O2B	0.107 (2)	0.0956 (19)	0.0407 (11)	−0.0337 (16)	−0.0147 (12)	−0.0019 (12)
O3B	0.0960 (18)	0.0621 (14)	0.0461 (11)	0.0021 (13)	−0.0096 (11)	0.0156 (10)
O4B	0.0511 (12)	0.122 (2)	0.0466 (11)	−0.0224 (13)	−0.0071 (9)	−0.0004 (13)
O1W	0.076 (2)	0.0548 (17)	0.0435 (15)	0.000	−0.0078 (14)	0.000

### Geometric parameters (Å, º)

**Table:**

Cr1A—N6A	1.980 (2)	C3A—H3A2	0.9800
Cr1A—N5A	1.989 (2)	C4A—C5A	1.512 (4)
Cr1A—N1A	2.080 (2)	C4A—H4A1	0.9800
Cr1A—N4A	2.0829 (19)	C4A—H4A2	0.9800
Cr1A—N3A	2.086 (2)	C5A—H5A1	0.9800
Cr1A—N2A	2.097 (2)	C5A—H5A2	0.9800
S1A—C11A	1.612 (2)	C6A—C7A	1.519 (4)
S2A—C12A	1.590 (2)	C6A—H6A1	0.9800
N1A—C1A	1.487 (3)	C6A—H6A2	0.9800
N1A—C10A	1.493 (3)	C7A—H7A1	0.9800
N1A—H1A	0.9900	C7A—H7A2	0.9800
N2A—C3A	1.492 (3)	C8A—C9A	1.502 (4)
N2A—C2A	1.502 (4)	C8A—H8A1	0.9800
N2A—H2A	0.9900	C8A—H8A2	0.9800
N3A—C6A	1.490 (3)	C9A—C10A	1.522 (4)
N3A—C5A	1.491 (4)	C9A—H9A1	0.9800
N3A—H3A	0.9900	C9A—H9A2	0.9800
N4A—C7A	1.490 (3)	C10A—H10A	0.9800
N4A—C8A	1.496 (3)	C10A—H10B	0.9800
N4A—H4A	0.9900	Cr2B—O2B	1.596 (2)
N5A—C11A	1.165 (3)	Cr2B—O3B	1.603 (2)
N6A—C12A	1.158 (3)	Cr2B—O4B	1.612 (2)
C1A—C2A	1.493 (4)	Cr2B—O1B1	1.746 (9)
C1A—H1A1	0.9800	Cr2B—O1B2	1.746 (9)
C1A—H1A2	0.9800	Cr2B—O1B1^i^	1.791 (9)
C2A—H2A1	0.9800	O1B1—O1B1^i^	0.686 (9)
C2A—H2A2	0.9800	O1W—H1OW	0.839 (7)
C3A—C4A	1.511 (5)	O1W—H1OW^ii^	0.839 (7)
C3A—H3A1	0.9800		
			
N6A—Cr1A—N5A	88.66 (9)	C5A—C4A—H4A1	108.5
N6A—Cr1A—N1A	92.76 (9)	C3A—C4A—H4A2	108.5
N5A—Cr1A—N1A	96.39 (9)	C5A—C4A—H4A2	108.5
N6A—Cr1A—N4A	175.72 (8)	H4A1—C4A—H4A2	107.5
N5A—Cr1A—N4A	87.44 (8)	N3A—C5A—C4A	114.4 (2)
N1A—Cr1A—N4A	89.43 (8)	N3A—C5A—H5A1	108.7
N6A—Cr1A—N3A	94.53 (9)	C4A—C5A—H5A1	108.7
N5A—Cr1A—N3A	92.73 (9)	N3A—C5A—H5A2	108.7
N1A—Cr1A—N3A	168.45 (8)	C4A—C5A—H5A2	108.7
N4A—Cr1A—N3A	83.89 (8)	H5A1—C5A—H5A2	107.6
N6A—Cr1A—N2A	87.88 (8)	N3A—C6A—C7A	108.5 (2)
N5A—Cr1A—N2A	176.30 (9)	N3A—C6A—H6A1	110.0
N1A—Cr1A—N2A	82.47 (8)	C7A—C6A—H6A1	110.0
N4A—Cr1A—N2A	96.05 (8)	N3A—C6A—H6A2	110.0
N3A—Cr1A—N2A	88.86 (9)	C7A—C6A—H6A2	110.0
C1A—N1A—C10A	112.12 (19)	H6A1—C6A—H6A2	108.4
C1A—N1A—Cr1A	109.52 (16)	N4A—C7A—C6A	107.9 (2)
C10A—N1A—Cr1A	116.98 (17)	N4A—C7A—H7A1	110.1
C1A—N1A—H1A	105.8	C6A—C7A—H7A1	110.1
C10A—N1A—H1A	105.8	N4A—C7A—H7A2	110.1
Cr1A—N1A—H1A	105.8	C6A—C7A—H7A2	110.1
C3A—N2A—C2A	109.8 (2)	H7A1—C7A—H7A2	108.4
C3A—N2A—Cr1A	115.22 (16)	N4A—C8A—C9A	112.3 (2)
C2A—N2A—Cr1A	107.01 (16)	N4A—C8A—H8A1	109.1
C3A—N2A—H2A	108.2	C9A—C8A—H8A1	109.1
C2A—N2A—H2A	108.2	N4A—C8A—H8A2	109.1
Cr1A—N2A—H2A	108.2	C9A—C8A—H8A2	109.1
C6A—N3A—C5A	112.4 (2)	H8A1—C8A—H8A2	107.9
C6A—N3A—Cr1A	107.93 (15)	C8A—C9A—C10A	116.0 (2)
C5A—N3A—Cr1A	118.5 (2)	C8A—C9A—H9A1	108.3
C6A—N3A—H3A	105.7	C10A—C9A—H9A1	108.3
C5A—N3A—H3A	105.7	C8A—C9A—H9A2	108.3
Cr1A—N3A—H3A	105.7	C10A—C9A—H9A2	108.3
C7A—N4A—C8A	110.21 (19)	H9A1—C9A—H9A2	107.4
C7A—N4A—Cr1A	106.58 (14)	N1A—C10A—C9A	113.6 (2)
C8A—N4A—Cr1A	116.75 (15)	N1A—C10A—H10A	108.8
C7A—N4A—H4A	107.7	C9A—C10A—H10A	108.8
C8A—N4A—H4A	107.7	N1A—C10A—H10B	108.8
Cr1A—N4A—H4A	107.7	C9A—C10A—H10B	108.8
C11A—N5A—Cr1A	170.5 (2)	H10A—C10A—H10B	107.7
C12A—N6A—Cr1A	176.3 (2)	N5A—C11A—S1A	178.8 (2)
N1A—C1A—C2A	107.5 (2)	N6A—C12A—S2A	179.0 (3)
N1A—C1A—H1A1	110.2	O2B—Cr2B—O3B	111.73 (13)
C2A—C1A—H1A1	110.2	O2B—Cr2B—O4B	109.44 (13)
N1A—C1A—H1A2	110.2	O3B—Cr2B—O4B	108.17 (13)
C2A—C1A—H1A2	110.2	O2B—Cr2B—O1B1	97.9 (2)
H1A1—C1A—H1A2	108.5	O3B—Cr2B—O1B1	111.5 (4)
C1A—C2A—N2A	108.3 (2)	O4B—Cr2B—O1B1	117.8 (3)
C1A—C2A—H2A1	110.0	O2B—Cr2B—O1B2	97.9 (2)
N2A—C2A—H2A1	110.0	O3B—Cr2B—O1B2	111.5 (4)
C1A—C2A—H2A2	110.0	O4B—Cr2B—O1B2	117.8 (3)
N2A—C2A—H2A2	110.0	O2B—Cr2B—O1B1^i^	119.5 (2)
H2A1—C2A—H2A2	108.4	O3B—Cr2B—O1B1^i^	104.8 (4)
N2A—C3A—C4A	111.4 (2)	O4B—Cr2B—O1B1^i^	102.3 (3)
N2A—C3A—H3A1	109.3	O1B1—Cr2B—O1B1^i^	22.3 (3)
C4A—C3A—H3A1	109.3	O1B2—Cr2B—O1B1^i^	22.3 (3)
N2A—C3A—H3A2	109.3	O1B1^i^—O1B1—Cr2B	82.5 (15)
C4A—C3A—H3A2	109.3	O1B1^i^—O1B1—Cr2B^i^	75.2 (15)
H3A1—C3A—H3A2	108.0	Cr2B—O1B1—Cr2B^i^	157.7 (3)
C3A—C4A—C5A	115.1 (2)	Cr2B—O1B2—Cr2B^i^	157.7 (3)
C3A—C4A—H4A1	108.5	H1OW—O1W—H1OW^ii^	109 (2)
			
C10A—N1A—C1A—C2A	171.4 (2)	C7A—N4A—C8A—C9A	176.9 (2)
Cr1A—N1A—C1A—C2A	39.8 (3)	Cr1A—N4A—C8A—C9A	−61.4 (2)
N1A—C1A—C2A—N2A	−55.7 (3)	N4A—C8A—C9A—C10A	65.3 (3)
C3A—N2A—C2A—C1A	169.5 (2)	C1A—N1A—C10A—C9A	−69.4 (3)
Cr1A—N2A—C2A—C1A	43.8 (2)	Cr1A—N1A—C10A—C9A	58.4 (3)
C2A—N2A—C3A—C4A	173.1 (2)	C8A—C9A—C10A—N1A	−64.1 (3)
Cr1A—N2A—C3A—C4A	−66.0 (3)	O2B—Cr2B—O1B1—O1B1^i^	166.3 (18)
N2A—C3A—C4A—C5A	68.6 (3)	O3B—Cr2B—O1B1—O1B1^i^	−76.5 (19)
C6A—N3A—C5A—C4A	−72.0 (3)	O4B—Cr2B—O1B1—O1B1^i^	49 (2)
Cr1A—N3A—C5A—C4A	55.0 (3)	O2B—Cr2B—O1B1—Cr2B^i^	166.3 (18)
C3A—C4A—C5A—N3A	−62.5 (4)	O3B—Cr2B—O1B1—Cr2B^i^	−76.5 (19)
C5A—N3A—C6A—C7A	170.2 (2)	O4B—Cr2B—O1B1—Cr2B^i^	49 (2)
Cr1A—N3A—C6A—C7A	37.7 (3)	O1B1^i^—Cr2B—O1B1—Cr2B^i^	0.004 (6)
C8A—N4A—C7A—C6A	172.4 (2)	O2B—Cr2B—O1B2—Cr2B^i^	166.3 (18)
Cr1A—N4A—C7A—C6A	44.8 (2)	O3B—Cr2B—O1B2—Cr2B^i^	−76.5 (19)
N3A—C6A—C7A—N4A	−55.9 (3)	O4B—Cr2B—O1B2—Cr2B^i^	49 (2)

Symmetry codes: (i) −*x*+1, −*y*+1, −*z*+1; (ii) −*x*+1, *y*, −*z*+3/2.

### Hydrogen-bond geometry (Å, º)

**Table:**

*D*—H···*A*	*D*—H	H···*A*	*D*···*A*	*D*—H···*A*
N1*A*—H1*A*···O1*W*^iii^	0.99	2.15	3.089 (3)	157
N2*A*—H2*A*···O3*B*	0.99	2.17	3.127 (3)	163
N3*A*—H3*A*···O4*B*^iv^	0.99	2.10	2.953 (3)	143
N4*A*—H4*A*···O4*B*	0.99	1.99	2.904 (3)	152
O1*W*—H1*OW*···O2*B*	0.84 (1)	2.24 (1)	3.052 (3)	164 (2)

Symmetry codes: (iii) −*x*+1/2, −*y*+3/2, −*z*+1; (iv) *x*, −*y*+1, *z*−1/2.
